# Cilostazole versus clopidogrel in acute large-vessel moderate and moderate-to-severe ischemic stroke: a randomized controlled trial

**DOI:** 10.1007/s10072-025-08107-9

**Published:** 2025-04-15

**Authors:** Sherihan Rezk ahmed, Mohamed Fouad elsayed Khalil, Mohamed Ismaiel, Tarek Youssif Omar, Hossam Mohamed Refat, Ahmed Ahmed Mohamed Kamal Ebied, Noha Abdelwahed, Ahmed Zaki Omar Akl, Emad Labib Abdelhamid Mahmoud, Salah Ibrahim Ahmed, Asmaa Mohammed Hassan, Islam Fathallah Mohamed Kamel, Amir Ahmed Elsaeed Egila, Mohamed Abouelnaga, Mohamed G. Zeinhom

**Affiliations:** 1 Neurology Department, Faculty of Medicine, Kafr El-Sheikh University, Kafr El-Sheikh, Egypt; 2 Neurology Department, NMC Royal Hospital, MBZ, Abu Dhabi, United Arab Emirates; 3 Neurology Department, Al-Sahel Teaching Hospital, Cairo, Egypt; 4 Neurology Department, Burjeel Medical Centers, Abu Dhabi, United Arab Emirates; 5 Neurology Department, Faculty of Medicine Zagazig University, Zagazig, Egypt; 6 Neurology Department, Emirates Hospital Group, Dubai, UAE; 7 Neurology Department, Faculty of Medicine, Ain Shams University, Cairo, Egypt; 8 Cardiology Department, National Heart Institute, Cairo, Egypt; 9 Neurology Department, Faculty of Medicine, Al-Azhar University, Assiut, Egypt; 10 Neurology Department, Medeor Hospital, Abu Dhabi, UAE; 11 Neurology Department, Faculty of Medicine, Tanta University, Tanta, Egypt; 12 Neurology Department, Security Forces Hospital, Riyadh, Saudi Arabia; 13 Neurology Department, Faculty of Medicine, Alexandria University, Alexandria, Egypt

**Keywords:** Cilostazol, Egypt, Clopidogrel, Large-vessel stroke, Moderate ischemic stroke, Moderate-to-severe stroke

## Abstract

**Background:**

More than one-third of all ischemic strokes are induced by large vessel occlusion (LVO). All the wide-scale trials that assessed the impacts of cilostazol versus clopidogrel in stroke management have been conducted in Asia and involved patients with minor stroke or TIA. Our trial is the first-ever study to evaluate cilostazol versus clopidogrel in acute LVO with moderate to severe ischemic stroke in North Africa.

**Objectives:**

We assessed the efficacy and safety of cilostazol versus clopidogrel in first-ever LVO moderate and moderate to severe ischemic stroke patients.

**Methods:**

580 moderate and moderate-to-severe LVO ischemic stroke participants were randomly enrolled to receive loading and maintenance doses of cilostazol or clopidogrel.

**Results:**

580 patients were included in the intention-to-treat analysis. 29 (10.0%) participants in the cilostazol arm and 43 (14.8%) participants in the clopidogrel arm experienced a new stroke (HR 0.37; 95% CI, 0.29–0.73; *P*-value = 0.03). Eight participants (2.8%) in the cilostazol arm and 17 patients (5.9%) in the clopidogrel arm had drug-related hemorrhagic complications (HR 0.29; 95% CI, 0.18–0.63; *P*-value = 0.008).

**Conclusion:**

Patients who experienced acute LVO moderate and moderate-to-severe ischemic stroke and received loading and maintenance doses of cilostazol within the first 24 h after stroke onset had better clinical outcomes based on recurrent stroke rates and better safety outcomes regarding hemorrhagic transformation of brain infarction and drug-induced peripheral hemorrhagic side effects compared to those who received loading and maintenance doses of clopidogrel. There were no significant differences between the two groups regarding death due to vascular events and unfavorable mRS after three months of stroke onset.

**Registration:**

Retrospectively registered on ClinicalTrials.gov, NCT06242145, 27–01-2024.

**Supplementary Information:**

The online version contains supplementary material available at 10.1007/s10072-025-08107-9.

## Background

More than one-third of all ischemic strokes are induced by large vessel occlusion (LVO); the term (LVO) describes the occlusion of the intracranial part of the internal carotid artery, proximal, middle cerebral arteries, intracranial vertebral artery (V.A.), and basilar artery (B.A.) [1].

Many studies showed that stroke associated with LVO was linked to poor functional outcomes, higher rates of recurrent stroke, a higher percentage of haemorrhagic transformation, and higher mortality rates when compared with non-LVO stroke [2, 3].

Many recent Guidelines recommend using antiplatelet agents to prevent post-stroke vascular events such as recurrent ischemic stroke and myocardial infarction [4, 5].

Cilostazol is a cyclic AMP phosphodiesterase inhibitor that elevates the cyclic adenosine monophosphate (AMP) levels in platelets, leading to the prevention of the platelet aggregation upon release of adenosine diphosphate (ADP), collagen, and epinephrine, and arachidonic acid [6]. Moreover, cilostazol induces many beneficial effects besides its antiplatelet action, such as vascular protection, antiproliferation, alleviation of ischemia–reperfusion injury, and antiatherosclerosis [7].

Clopidogrel is an irreversible inhibitor of ADP that prevents its binding to platelet P2Y12 receptor and activates the glycoprotein IIb/IIIa complex, leading to the prevention of platelets sticking together in acute ischemic events [8].

When cilostazol was compared with aspirin and ticlopidine, it showed a comparable ability to inhibit platelet reactivity and aggregation to that produced by ticlopidine and aspirin. Also, cilostazol produced fewer hemorrhagic side effects [9, 10].

All the randomized large clinical trials that assessed the efficacy and safety of cilostazol in stroke prevention have been conducted in Asia and included patients with acute minor stroke or TIA; moreover, none of these trials evaluated the potential role of cilostazol in LVO moderate or moderate-to-severe ischemic stroke [11]. Thus, we aimed to evaluate the safety and efficacy of cilostazol versus clopidogrel in first-ever non-cardioembolic LVO moderate and moderate to severe ischemic stroke patients.

## Methods

### Trial design

We evaluated the safety and efficacy of cilostazol in comparison with clopidogrel in patients experiencing a first-ever LVO moderate and moderate to severe ischemic stroke.

We got the acceptance of the Kafr el-Sheikh University Hospital ethical committee.

We evaluated all LVO moderate and moderate-to-severe AIS patients presented to Kafr-Elsheikh University Hospital, Al Obour Insurance Hospital in Kafr Elsheikh between 1st September 2022 and 1st September 2024; the last patient joined the trial on 25th July 2024.

We prepared our randomization plan using a computer program; the block size was four, and the allocation sequences were repeated within a fixed block length of four, so the two treatments, A and B, had six block sequences, which were (AABB, BBAA, ABAB, BABA, ABBA, BAAB.

Our patients received cilostazol or clopidogrel in a 1:1 ratio during the first 24 h after stroke onset.

### Participants

Our study included male and female participants who experienced acute first-ever large-vessel moderate or moderate-to-severe ischemic stroke and were ineligible to receive alteplase. Our study had two parallel groups: the (A) group, which involved 290 patients who received cilostazol, and the (B) group, which involved 290 patients who received clopidogrel.

### Eligibility criteria

#### Inclusion criteria

Our study involved participants aged between 18 and 75 years who experienced their acute first-ever LVO moderate or moderate-to-severe ischemic stroke. We considered stroke as moderate when the patients' NIHSS ranged from five to fifteen and considered stroke as moderate to severe if patients' NIHSS ranged from sixteen to twenty [12, 13].

We diagnosed LVO stroke according to TOAST classification if 1- patient had clinical and brain imaging findings of > 50% stenosis or occlusion of at least one of the following arterial segments on computed tomography angiography (CTA) or magnetic resonance angiography (MRA) if CTA was contraindicated: intracranial portion of internal carotid arteries, middle cerebral arteries (M1/M2), the intracranial portion of vertebral arteries, and basilar artery, 2- the patient had cortical or cerebellar lesions and brain stem or subcortical hemispheric infarcts greater than 1.5 cm in diameter on CT or MRI, 3- there were no potential sources of cardiogenic embolism [14].

Our patients should start antiplatelet agents within the first 24 h of stroke symptoms; our study did not include participants older than 75 years as advancing age is associated with an increased risk of hemorrhagic transformation (HT), as well as worse stroke outcomes. Older patients experience an increase in systemic inflammation and blood–brain barrier (BBB) permeability and have a more significant burden of cerebrovascular disease, hypertension, and diabetes, which induce inflammation and atherosclerosis, so if a stroke occurs, the age-related inflammation produces blood–brain barrier disruption leading to an increased risk of HT [15, 16] and the increased risk of HT in those patients might cloud our safety outcome analysis. Also, patients older than 75 years old have a higher possibility of atrial fibrillation, which necessitates anticoagulation, which was a contraindication in our study [17]. Our study involved patients who experienced previous TIA but did not involve patients who received alteplase or underwent thrombectomy to prevent clouding safety assessment [18].

#### Exclusion criteria

We excluded patients who received alteplase or underwent arterial revascularization during the first seven days of the study as alteplase and endovascular thrombectomy might lead to hemorrhagic complications and bias our assessment of the safety of the trial medications. Also, in patients who are treated with alteplase, initiation of antiplatelet agents should be delayed until after 24 h post-thrombolysis [19], while our trial included patients who received antiplatelet within the first 24 h of stroke onset, and the median time to antiplatelet starting was 17 h with more than 75% of our patients receiving treatment within the first 20 h of stroke. Additionally, most of the large-scale randomized trials that evaluated the safety and efficacy of different antiplatelets excluded patients who received alteplase or underwent thrombectomy to avoid clouding the safety analysis [9–11]. Our study did not involve participants who were administered antiplatelets or anticoagulants during the last three days before enrolment in the trial to avoid clouding safety assessment [18]. In addition, we did not include participants whose NIHSS was less than four or more than 20 and patients who experienced disorders linked to recurrent neurological deficits, such as epilepsy and multiple sclerosis. We ruled out patients who experienced cardiological disorders preventing using cilostazol as unstable angina, myocardial infarction, or heart failure, which was diagnosed when ejection fraction was < 40% at echocardiography [20], as cilostazol has a positive inotropic effect and increased in mortality rate in patients with heart failure [21].

We did not include patients who experienced a cardioembolic stroke. We diagnosed cardioembolic strokes when the participant had major or minor risk factors of a potential cardiac source of emboli such as mechanical cardiac valves, atrial fibrillation (AF), and patent foramen ovale [22, 23]. We determined AF if there was a minimum of 30 s of cardiac rhythm, demonstrating the absence of P waves and irregular RR intervals (when atrioventricular conduction was not impaired) in a conventional 12-lead ECG recording [24].

We excluded regular users of medications affecting cilostazol metabolism, such as macrolide antibiotics, diltiazem, omeprazole, fluoxetine, and sertraline [25] or regular users of drugs influencing clopidogrel metabolism, such as proton pump inhibitors, statins, ketoconazole, and rifampin [26].

We ruled out participants who experienced recurrent ischemic stroke diagnosed using their clinical data and/or MRI brain findings. In addition, we excluded participants who experienced hypersensitivity to cilostazol or clopidogrel and patients who had INR greater than 1.4, PT more than 18 s, or Platelets count < 100,000 platelets per microliter.

We ruled out participants who experienced organ failure such as renal failure and liver cell failure, active malignancies, and patients with active peptic ulcer bleeding history within the last year.

We excluded pregnant and lactating patients, patients with cerebral venous thrombosis, and stroke associated with cardiac arrest.

We did not include patients with stenosis or occlusion of the cerebral posterior artery as patients with stenosis in the branch cortical artery and first portion of cerebral posterior artery do not fulfill the TOAST classification of LVO stroke [14].

## Interventions

Our study involved two groups. The (A) arm, which included 290 patients who were administered a 200 mg loading dose of cilostazol during the first 24 h of LVO stroke, then continued on 100 mg twice daily till the 90th day after LVO stroke, and the (B) arm, which involved 290 patients who were administered a 300 mg loading dose of clopidogrel during the first 24 h of LVO stroke, then continued on 75 mg per day till the 90th day after LVO stroke.

We collected the complete clinical data of our participants, including the relevant clinical history and examination findings; every patient in our trial underwent CT and MRI brain imaging, including T1W, T2W, FLAIR, DWI, T2 Echo Gradient, CTA or MRA (if CTA was contraindicated) of brain and carotid vessels to diagnose LVO stroke and exclude haemorrhagic stroke; moreover, all of our patients underwent a follow-up CT brain during the seven days of stroke onset to detect hemorrhagic infarction and were classified according to the European Cooperative Acute Stroke Study (ECASS) classification [27].

Our patients underwent a 12-lead routine ECG and transthoracic echocardiography (TTE) to detect heart failure, myocardial infarction, atrial fibrillation, and valvular heart diseases before being enrolled in our trial.

After our participants were enrolled in one of the two groups and were administered loading cilostazol or clopidogrel, they underwent continuous ECG monitoring for one day. We found that five patients in the cilostazol group and three patients in the clopidogrel group had atrial fibrillation. Those patients stopped antiplatelet agents prematurely, started anticoagulant therapy [28], and were included in the intention-to-treat analysis.

All the patients had an evaluation of their complete blood count, coagulation profile, lipid profiles, liver functions, and blood glucose level and we diagnosed diabetes when fasting plasma glucose level was more than 126 mg/dl, or casual plasma glucose was more than 200 mg/dl, or HbA1C was more than 6.5 [29, 30].

We assessed blood pressure and hypertension was diagnosed according to the 2020 International Society of Hypertension Practice Guidelines when systolic blood pressure was more than 140 mmHg and/ or diastolic blood pressure was more than 90 mm/Hg in two or more different office visit measurements [31, 32].

In our study, the follow-up period was 90 days, during which we telephoned our patients twice a week and met them in our hospital once a month. However, if any patient complained of features consistent with the possibility of recurrent stroke during the follow-up period, we directed him to our hospital to be evaluated.

## Outcomes

### The primary outcomes

The primary efficacy outcome was the rate of new stroke (either ischemic or hemorrhagic) within ninety days post-stroke [33], while the primary safety outcome was the rate of treatment-related haemorrhagic complications evaluated using the PLATO bleeding definition [34].

### The secondary outcomes

In our study, there were three secondary efficacy endpoints: the percentage of patients presenting with a composite of myocardial infarction, new stroke, and death due to vascular events during three months after stroke onset, the percentage of participants presenting with recurrent ischemic stroke, and the rate of participants who had an unfavorable outcome (modified Rankin scale (mRS) more than two) [35, 36] within three months, while the secondary safety endpoints were two points; the first was the rate of participants who suffered from a hemorrhagic transformation of brain infarction, and we defined Symptomatic hemorrhagic transformation of brain infarction as parenchymal hematoma (PH) occurring following ischemic stroke and causing an increase in NIHSS by 4 points or more [37] the second was the rate of participants who suffered from treatment-related side effects assessed via questionnaire.

### Sample size

We utilized Power Analysis & Sample Size System (PASS, V12), NCSS) to identify our sample size and detected that 526 large-vessel ischemic stroke patients would provide 80% power to detect a relative risk reduction of 35% in new ischemic stroke (primary outcome) in the cilostazol group as compared with the clopidogrel group, with a final two-sided significance level of 95%, alpha error of 5%, assuming an incidence of new ischemic stroke of 13.6% [38] in the clopidogrel group and an overall dropout rate of 5%. The final size of our trial was 580 patients, 290 patients in each group.

### Randomization and blinding

Before enrolling participants in a group, we obtained signed informed consent from the participants or their siblings. The trial was blinded to the investigators; a separate statistician provided us with a computer-based four-block randomization plan; we randomly enrolled participants in a one-to-one ratio to administer cilostazol or clopidogrel by a specially trained nurse. Our investigators were not aware of antiplatelet administration. We prepared consecutively numbered opaque closed envelopes and 580 labels for each drug labeled Drug A or B. Following our randomization plan, we sorted the labels into envelopes numbered 1 to 580. The numbers assigned to patients began from one to 580 and were documented in the medical records. Then, we opened the files that matched the patient enrollment number, and according to the randomization, the patient was assigned to be administered treatment A or B. Treatment A included cilostazol 100 mg tablets, and treatment B included clopidogrel 75 mg tablets. An independent blinded statistician performed our statistical analysis. Our trial did not involve using a placebo due to the unavailability of funding.

On the other hand, our participants were instructed to refrain from telling their physician about the treatment and to inform the nurse. The nurse would inform the physician if participants suffered medication-related side effects. A consultant neurologist was responsible for the follow-up visits, and a specially-trained nurse handled the follow-up calls.

### Statistical analysis of the data

The two-sided analysis of our data was performed by an independent statistician using the IBM SPSS software package, version 20.0 (Armonk, NY: IBM Corp.), and the analysis of safety and efficacy endpoints depended on the intention-to-treat principle. We used the Shapiro–Wilk test to assess the distribution of the quantitative data. We expressed them as means ± S.D. if they were normally distributed or median and interquartile range (IQR) if they were abnormally distributed. We utilized the Mann–Whitney U test to compare the abnormally distributed quantitative data and Pearson's chi-square to compare the qualitative data. We determined statistically significant variations between the two groups if the *P*-value was less than 0.05. As we have more than one secondary efficacy endpoint, we utilized Bonferroni correction to avoid type 1 statistical error, and we determined statistically significant variations between the two groups if the *P*-value was less than 0.0167. We performed the Kaplan–Meier test to evaluate the survival analysis and the log-rank technique to evaluate the antiplatelet's role in different endpoints' occurrence. At a 95% confidence interval (CI), the hazard ratio (H.R.) was calculated using the Cox regression method. H.R. was considered significant when one does not lie between lower and upper CI. We adjusted the following confounding factors in the Cox regression model: age, smoking, dyslipidemia, diabetes mellitus, ischemic heart disease, TOAST classification of stroke, previous TIA, number of prior antiplatelet, number of prior statins, gender, and type of circulation affected.

## Results

**Fig. 1:**
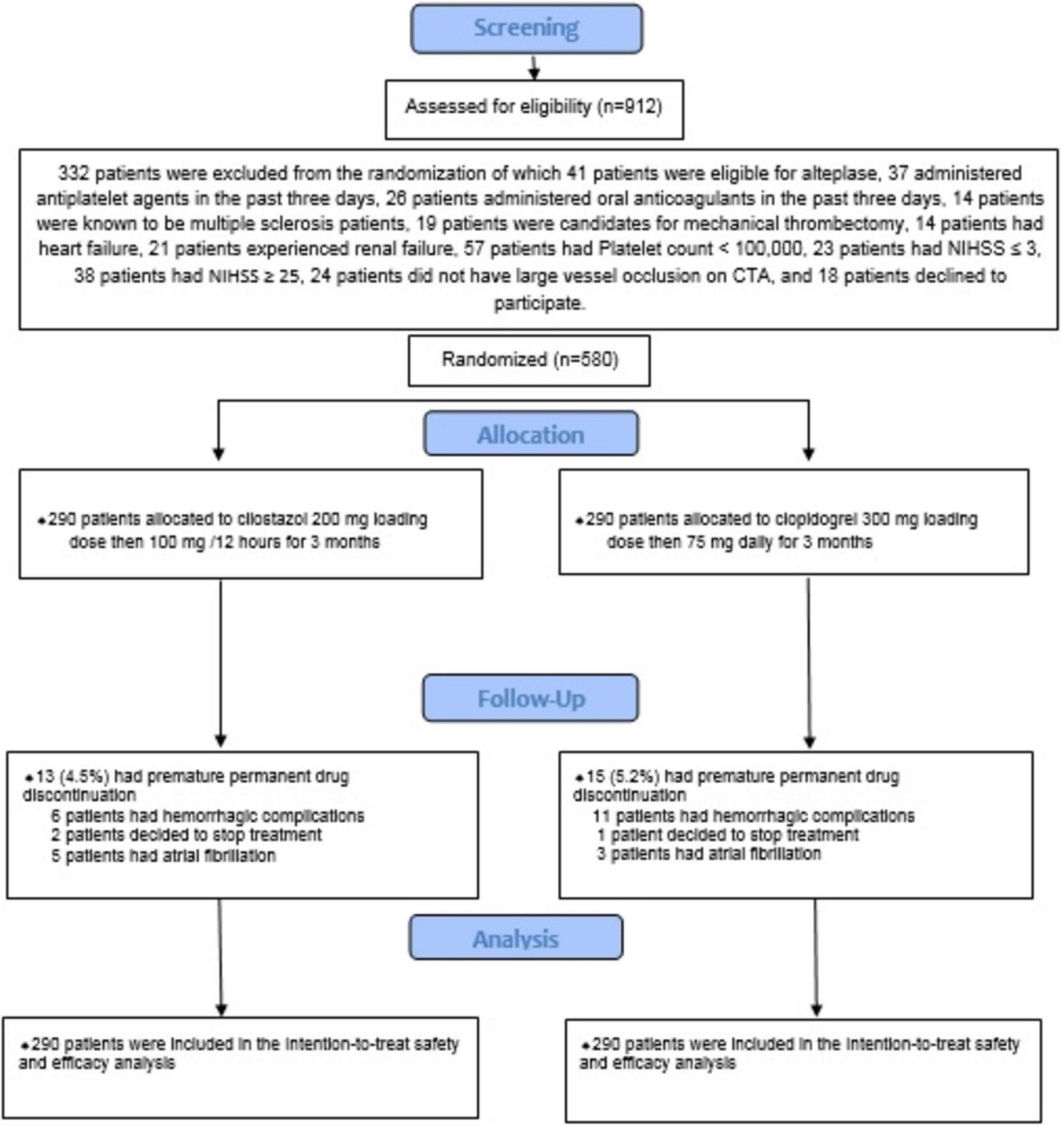
Study flow diagram

We evaluated 912 patients who experienced their first-ever acute LVO ischemic stroke to establish their eligibility to be enrolled in our study. Eventually, 580 patients (349 males and 231 females) were enrolled to be administered cilostazol or clopidogrel, as shown in Fig. 1.

In our trial, no statistically relevant differences existed in the characteristics of the two patient groups, as shown in Table 1.

**Table 1:** Baseline characteristics of all participants

Character	Cilostazol group (*n* = 290)	Clopidogrel group (*n* = 290)	*P*-value
Age, no. (percentage) *			
18 – 27, no. (percentage)	**14.0 (4.8%)**	**11.0 (3.8%)**	**0.55**
28 – 37, no. (percentage)	**19.0 (6.6%)**	**21.0 (7.2%)**	
38 – 47, no. (percentage)	**46.0 (15.9%)**	**55.0 (19.0%)**	
48 – 57, no. (percentage)	**72.0 (24.8%)**	**66.0 (22.8%)**	
58 – 67, no. (percentage)	**64.0 (22.1%)**	**76.0 (26.2%)**	
68 – 75, no. (percentage)	**75.0 (25.9%)**	**61.0 (21.0%)**	
Male, no. (percent) *	**165.0 (56.9%)**	**184.0 (63.4%)**	**0.11**
Stroke severity, no. (percent) *			
Moderate stroke	**157**	**146**	**0.36**
Moderate-to-severe stroke	**133**	**144**	
Time till receiving treatment, hours median (IQR) †	**17.0 (14.0–20.0)**	**17.0 (15.0–19.0)**	**0.23**
Medical history, no. (percent) *			
Smoker	**194.0 (66.9%)**	**178.0 (61.4%)**	**0.17**
dyslipidemia	**201.0 (69.3%)**	**212.0 (73.1%)**	**0.31**
Diabetes mellites	**112.0 (38.6%)**	**103.0 (35.5%)**	**0.44**
Hypertension	**203.0 (70.0%)**	**207.0 (71.4%)**	**0.72**
IHD	**176.0 (60.7%)**	**182.0 (62.8%)**	**0.61**
Baseline NIHSS, median (IQR) †	**14.0 (13.0—20.0)**	**14.0 (12–20)**	**0.28**
Stroke vascular territory according to brain imaging*			
Middle cerebral artery	**127.0 (43.8%)**	**121.0 (41.7%)**	**0.78**
Vertebral	**19.0 (6.6%)**	**23.0 (7.9%)**	
Basilar	**61.0 (21.0%)**	**70.0 (24.1%)**	
Intracranial internal carotid	**73.0 (25.2%)**	**69.0 (23.8%)**	
More than one vessel	**10.0 (3.4%)**	**7.0 (2.4%)**	
Previous TIA, no. (percent) *	**54.0 (18.6%)**	**47.0 (16.2%)**	**0.44**
Number of prior antiplatelet, no. (percent) *	**23.0 (7.9%)**	**26.0 (9.0%)**	**0.65**
Number of prior statins, no. (percent) *	**17.0 (5.9%)**	**13.0 (4.5%)**	**0.45**
Anterior circulation stroke, no. (percent) *	**206.0 (71.0%)**	**197.0 (67.9%)**	**0.42**

^†^: median (interquartile range: IQR), *: percentage, IHD: Ischemic heart disease, TIA: transient ischemic attack

**Fig. 2:**
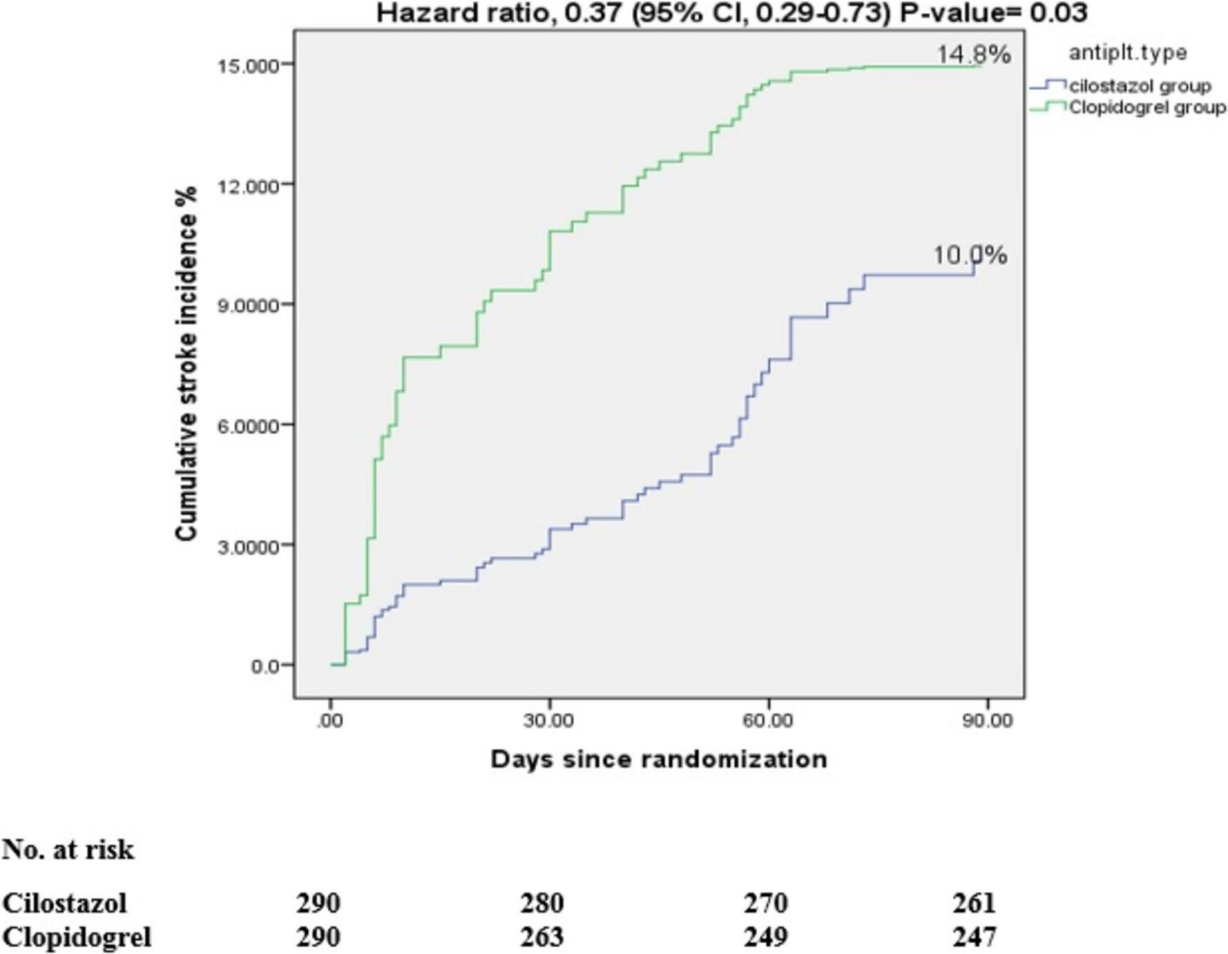
Cumulative incidence of any stroke in all patients at 90 days

29 (10.0%) participants in the cilostazol arm and 43 (14.8%) participants in the clopidogrel arm experienced a new stroke (hemorrhagic or ischemic) (HR 0.37; 95% CI, 0.29–0.73; *P*-value = 0.03), as shown in Table 2, Fig. 2.

**Table 2:** Analysis of efficacy and safety outcomes in all patients

Efficacy outcomes	Cilostazol group (*n* = 290)	Clopidogrel group (*n* = 290)	Hazard ratio (95% CI)	*P*-value
Primary efficacy outcome §				
New stroke	**29.0 (10.0%)**	**43.0 (14.8%)**	**0.37 (0.29–0.73)**	0.03**
Secondary efficacy outcomes ∥				
Composite of new stroke, MI, death	**40.0 (13.8%)**	**54.0 (18.6%)**	**0.41 (0.43–1.1)**	**0.09**
New ischemic stroke	**26.0 (9.0%)**	**36.0 (12.4%)**	**0.21 (0.51–1.09)**	**0.08**
Unfavorable outcome mRS more than 2 after 90 days	**173 (59.7%)**	**170.0 (58.6%)**	**0.54 (0.64–1.17)**	**0.16**
Primary safety outcome ¶				
Total Hemorrhagic complications	**8.0 (2.8%)**	**17.0 (5.9%)**	**0.29 (0.18–0.63)**	0.008**
Secondary safety outcomes ††				
Hemorrhagic transformation of infarction	**3.0 (1.0%)**	**7.0 (2.4%)**	**0.27 (0.31- 0.84)**	0.006**
Hemorrhagic infarction (HI) type 1 and 2	**2.0 (0.7%)**	**3.0 (1.0%)**	**0.73 (0.59–1.17)**	**0.51**
Parenchymal hematoma (PH) type 1 and 2	**1.0 (0.3%)**	**4.0 (1.4%)**	**0.22 (0.34–0.71)**	0.009**
Patients with non-hemorrhagic complications	**30.0 (10.3%)**	**28.0 (9.7%)**	**0.68 (0.47–1.32)**	**0.38**

^**§**^: Primary efficacy outcome, ∥: Secondary efficacy outcomes, ¶: Primary safety outcome, ††: Secondary safety outcomes, NIHSS: national institute of health stroke scale, mRS: modified Rankin scale, CI: confidence interval

^**^: Statistically significant at *P*-value < 0.05 for primary efficacy and primary safety outcomes, Statistically significant at adjusted *P*-value < 0.017 for secondary efficacy outcomes, and Statistically significant at adjusted *P*-value < 0.03 for secondary safety outcomes

The incidences of recurrent stroke, composite events outcomes, unfavourable outcome, hhemorrhagic complications, and non-hemorrhagic complications are Kaplan–Meier estimates of the percentage of patients with events at 90 days

26 (9.0%) participants in the cilostazol arm and 36 (12.4%) in the clopidogrel arm experienced a new ischemic stroke (HR 0.21; 95% CI, 0.51–1.09; *P*-value = 0.08). Moreover, 40 (13.8%) participants in the cilostazol arm and 54 (18.6%) in the clopidogrel arm experienced a composite of a new stroke, MI, or death due to vascular insults (HR 0.41; 95% CI, 0.43–1.1; *P*-value = 0.09). Finally, 173 (51.5%) participants in the cilostazol arm and 170 (57.9%) in the clopidogrel arm had a 3-month unfavorable mRS (HR 0.54; 95% CI, 0.64–1.17; *P*-value 0.16), as shown in Table 2.

**Fig. 3:**
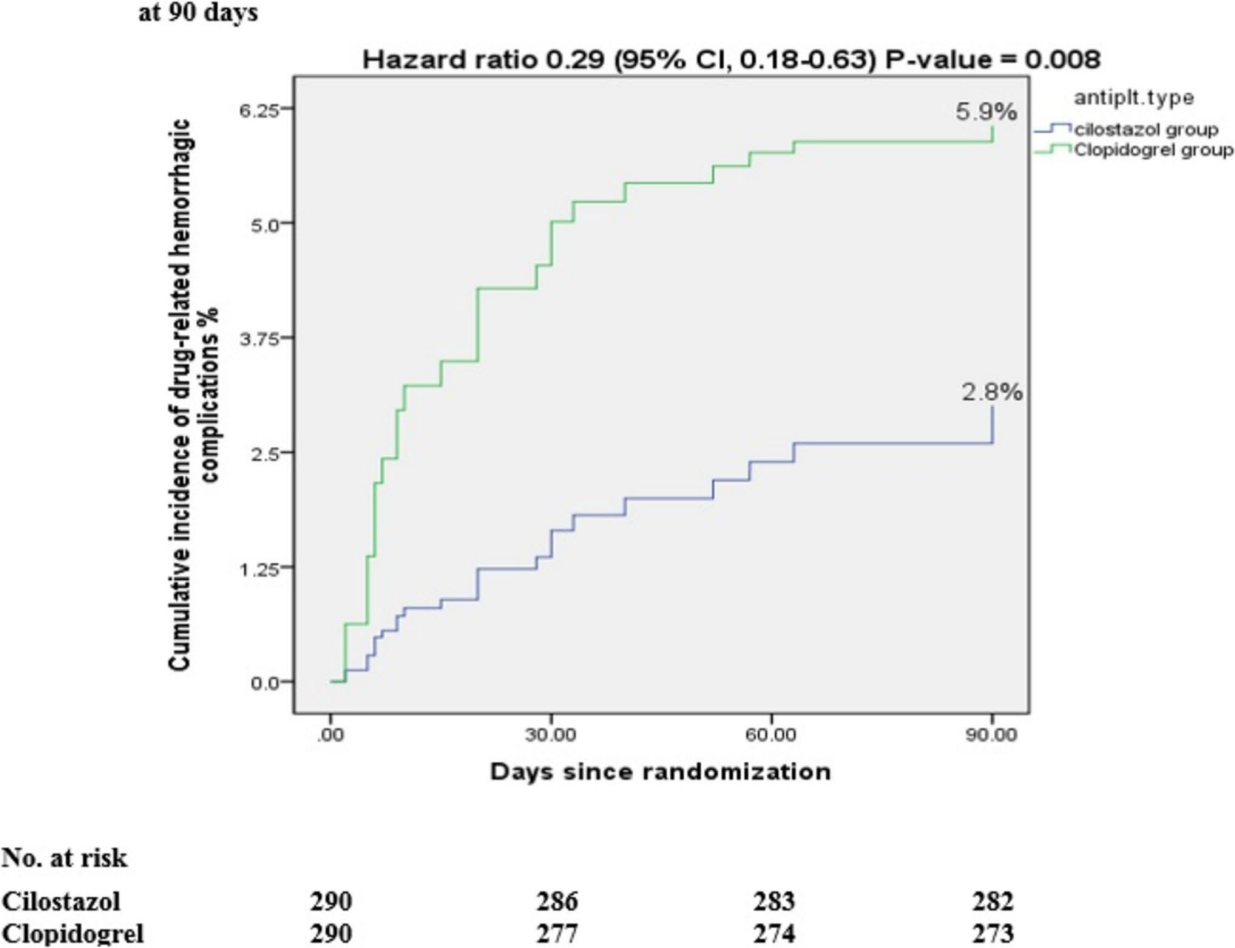
Cumulative incidence of drug-related hemorrhagic complications in all patients at 90 days

We also found that eight participants (2.8%) in the cilostazol arm suffered from drug-related hemorrhagic complications. Of them, three had minimal bleeding; two had minor bleeding and three experienced major bleeding. In the clopidogrel arm, 17 patients (5.9%) in the clopidogrel arm had drug-related hemorrhagic complications. Of them, seven had minimal bleeding, three had minor bleeding, and seven experienced major bleeding (HR 0.29; 95% CI, 0.18–0.63; *P*-value = 0.008), as shown in Table 2, and Fig. 3.

Thirty (10.3%) patients in the cilostazol group had drug-related non-hemorrhagic side effects. Of them, nine (3.1%) had headaches, five (1.7%) suffered from diarrhea, eight patients (2.8%) complained of palpitation, three (1.0%) had dizziness, six (2.1%) had tachycardia, and two (0.7%) had nausea and vomiting. In comparison, 28 (9.7%) patients in the clopidogrel group had drug-related non-hemorrhagic side effects. Of them, eight (2.8%) had nausea and vomiting, five patients (1.7%) had diarrhea, six (2.1%) suffered from back pain, three (1.0%) had chest tightness, and four (1.4%) complained of headache (HR 0.68; 95% CI, 0.47–1.32; *P*- value = 0.38), as shown in Table 2.

Two patients in the cilostazol group and one patient in the clopidogrel group decided to stop the treatment prematurely due to intolerable side effects, mainly headache and palpitation in the cilostazol group and nausea and vomiting in the clopidogrel group (HR 0.57; 95% CI, 0.38–1.27; *P*-value = 0.31).

**Fig. 4:**
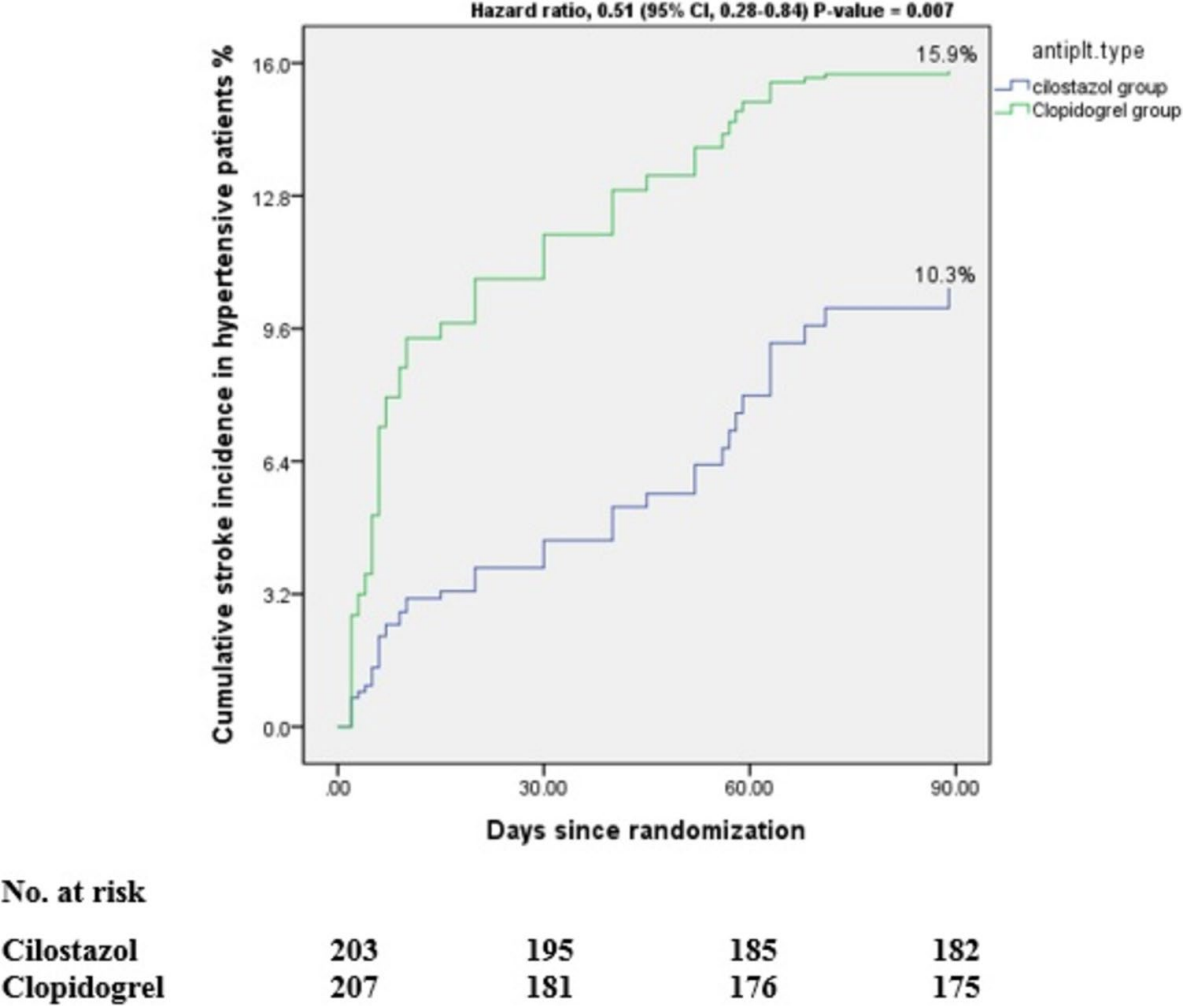
Cumulative incidence of new stroke in hypertensive patients at 90 days

When we analyzed the primary and secondary endpoints in hypertensive patients, we found that 21 (10.3%) participants in the cilostazol arm and 32 (15.9%) participants in the clopidogrel arm experienced new strokes (HR 0.51; 95% CI, 0.28–0.84; *P*-value = 0.007), as shown in Table 3 and Fig. 4.

**Table 3:** Analysis of efficacy and safety outcomes in hypertensive patients

Efficacy outcomes	Cilostazol group (*N* = 203)	Clopidogrel group (*N* = 207)	Hazard ratio (95% CI)	*P*-value
Primary efficacy outcome §				
New stroke	**21.0 (10.3%)**	**32.0 (15.9%)**	**0.51 (0.28–0.84)**	0.007**
Secondary efficacy outcomes ∥				
Composite of new stroke, MI, death	**29.0 (14.5%)**	**39.0 (18.8%)**	**1.16 (0.82–1.39)**	**0.12**
New ischemic stroke	**19.0 (9.4%)**	**27.0 (13%)**	**0.33 (0.54–1.07)**	**0.07**
Unfavorable outcome mRS more than 2 after 90 days	**121 (60.5%)**	**117.0 (56.5%)**	**0.83 (0.91–1.46)**	**0.21**
Primary safety outcome ¶				
Total Hemorrhagic complications	**6.0 (3.0%)**	**12.0 (5.8%)**	**0.26 (0.14–0.71)**	0.02**
Secondary safety outcomes ††				
Hemorrhagic transformation of infarction	**2.0 (1.0%)**	**5.0 (2.4%)**	**0.31 (0.18- 0.78)**	0.005**
Hemorrhagic infarction (HI) type 1 and 2	**1.0 (0.5%)**	**2.0 (1.0%)**	**0.66 (0.48–1.21)**	**0.32**
Parenchymal hematoma (PH) type 1 and 2	**1.0 (0.5%)**	**3.0 (1.4%)**	**0.31 (0.38–0.83)**	0.008**
Patients with non-hemorrhagic complications	**22.0 (10.8%)**	**20.0 (9.7%)**	**1.07 (0.52–1.19)**	**0.37**

^**§**^: Primary efficacy outcome, ∥: Secondary efficacy outcomes, ¶: Primary safety outcome, ††: Secondary safety outcomes, NIHSS: national institute of health stroke scale, mRS: modified Rankin scale, CI: confidence interval

^**^: Statistically significant at *P*-value < 0.05 for primary efficacy and primary safety outcomes, Statistically significant at adjusted *P*-value < 0.017 for secondary efficacy outcomes, and Statistically significant at adjusted *P*-value < 0.03 for secondary safety outcomes

The incidences of recurrent stroke, composite events outcomes, unfavourable outcome, hhemorrhagic complications, and non-hemorrhagic complications are Kaplan–Meier estimates of the percentage of patients with events at 90 days

We also found that 19 (9.4%) patients in the cilostazol arm and 27 (13.0%) in the clopidogrel arm experienced an ischemic stroke (HR 0.33; 95% CI, 0.54–1.07; *P*-value = 0.07). Moreover, 29 (14.5%) patients in the cilostazol arm and 39 (18.8%) in the clopidogrel arm experienced a composite of a new stroke, MI, or death due to vascular insults (HR 1.16; 95% CI, 0.82–1.39; *P*-value = 0.12). Finally, 121 (60.5%) patients in the cilostazol arm and 117 (56.5%) in the clopidogrel arm experienced a 3-month unfavorable mRS score (HR 0.83; 95% CI, 0.91–1.46; *P*-value 0.21), as shown in Table 3.

**Fig. 5:**
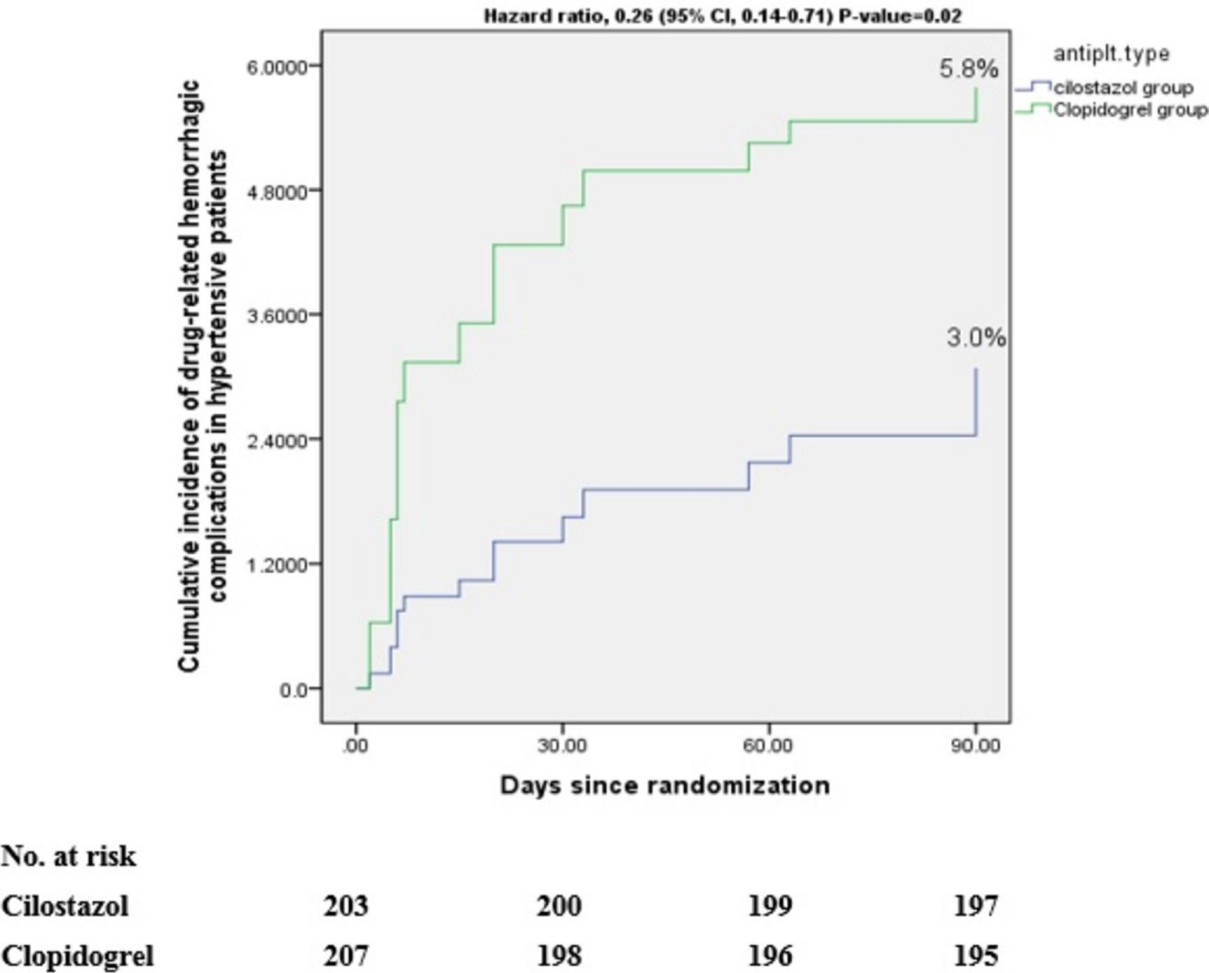
Cumulative incidence of drug-related hemorrhagic complications in hypertensive patients at 90 days

Six participants (3%) in the cilostazol arm suffered from drug-related hemorrhagic complications. Of them, three participants had minimal bleeding; one had minor bleeding, and two experienced major bleeding. In the clopidogrel group, 12 patients (5.8%) had drug-related hemorrhagic complications. Of them, five had minimal bleeding, two patients had minor bleeding, and five patients experienced major bleeding (HR 0.26; 95% CI, 0.14–0.71; *P*-value = 0.02), as shown in Table 3, Fig. 5.

Twenty-two (10.8%) patients in the cilostazol group had drug-related non-hemorrhagic side effects; of them, six (3.0%) had headache, three (1.5%) suffered from diarrhea, six (3.0%) complained of palpitation, two (1.0%) had dizziness, three (1.5%) had tachycardia, and two (1.0%) had nausea and vomiting. In comparison, twenty (9.7%) patients in the clopidogrel group had drug-related non-hemorrhagic side effects, of them, six (2.9%) had nausea and vomiting, three (1.4%) had diarrhea, five (2.4%) suffered back pain, two (1.0%) had chest tightness, and three (1.4%) complained of headache (HR 1.07; 95% CI, 0.52–1.19; *P*-value = 0.37), as shown in Table 3.

## Discussion

LVO induces more than one-third of all ischemic strokes. Moreover, LVO stroke leads to poor functional outcomes, higher rates of recurrent stroke, and higher mortality rates when compared with non-LVO stroke [39, 40].

Many previous studies [41–44] comparing the activity of cilostazol and aspirin found that they had a comparable ability to inhibit platelet reactivity and aggregation, so we aimed to evaluate a different hypothesis regarding the safety and efficacy of cilostazol versus clopidogrel in Egyptian patients who presented with LVO moderate and moderate-to-severe as we lack such studies in African patients.

Our study's main strength is that it was the first-ever trial worldwide to evaluate the safety and efficacy of cilostazol versus clopidogrel in acute LVO moderate and moderate-to-severe ischemic stroke patients from North Africa.

In our trial, the participants were administered a single antiplatelet after experiencing moderate or moderate-to-severe LVO stroke according to the guidelines [45]. Additionally, the subgroup analysis of CHANCE, CHARISMA, and MATCH trials showed that dual antiplatelet treatment in LVO stroke was associated with an increased risk of haemorrhagic complications [46–48].

Our patients did not undergo genetic analysis of the CYP2C19loss-of-function alleles, as the prevalence of CYP2C19loss-of-function among African and Caucasian patients is about 5% [49]. In addition, a genetic sub-study of the PLATO trial stated that another antiplatelet, ticagrelor, showed better efficacy in managing acute coronary syndromes than clopidogrel despite the CYP2C19 genotype [46].

Our trial showed that cilostazol administration after LVO stroke led to a reduction in the rate of composite of recurrent ischemic stroke, MI, and vascular complications mortality comparable to the reduction achieved after administrating clopidogrel, in agreement with the findings of other studies who found that cilostazol and clopidogrel showed approximate efficacy results in reducing recurrent ischemic stroke and mortality due to vascular event [50–52].

On the other hand, patients who were administered cilostazol experienced significantly fewer hemorrhagic strokes and peripheral hemorrhagic side effects in comparison with patients who were administered clopidogrel; these findings were in line with those of Liu et al., who found that administering cilostazol led to fewer hemorrhagic transformation of stroke, [7] whereas they are in contrast with the findings of Kwon et al. and Lee et al. [50, 51], who did not detect important changes in hemorrhagic infarction rates between cilostazol and clopidogrel groups.

The hypothesis was that cilostazol was able to decrease the rate of recurrent stroke and achieve lower rates of central and peripheral drug-induced hemorrhagic side effects in comparison with clopidogrel because it exerts its antiplatelet action by enhancing the endothelial cell function, decreasing activated platelets and preventing platelet aggregation induced by collagen and ADP while exerting minimal impact on the bleeding time. In contrast, clopidogrel exerts its antiplatelet action by permanently inhibiting the ADP P2Y12 receptor on platelets, leading to much longer bleeding time owing to irreversible inhibition of platelet aggregation [53–55].

Many trials showed that aspirin and clopidogrel significantly prolonged bleeding time in patients who experienced peripheral arterial diseases when used either individually or in combination, unlike cilostazol, which did not prolong the bleeding time significantly [53].

When we performed subgroup analysis, we found that hypertensive patients who received cilostazol had significantly lower rates of hemorrhagic stroke and drug-related hemorrhagic complications than patients receiving clopidogrel; our findings were in line with those of Lee et al., who found that cilostazol decreased the risk of recurrent stroke compared to clopidogrel partially due to the fact that, cilostazol produced vasodilation and suppressed angiotensin 2-induced hypertensive endothelial dysfunction leading to decreasing systolic blood pressure during the follow-up period and eventually enhanced protection against stroke recurrence [56].

Although our trial shows promising results, it has some limitations: first, our study was single-blinded; second, all our participants were Egyptian, which decreased the capability to evaluate outcomes of other ethnicities who had different genetic characteristics; third, our follow-up period was limited to three months, and this limited our abilities to monitor long-term outcomes; fourth, the findings in the hypertensive group were extracted from post hoc analysis, which was vulnerable to data dredging; fifth, we did not use placebo in our study as we did not have funds from our university or the pharmaceutical companies to manufacture placebo owing to the economic crisis in Egypt, which inhibited many pharmaceutical companies from sharing in clinical trials so, we need to perform a large, stratified, double-blinded study including patients from different ethnicities to establish the validity and generalizability of these findings.

## Conclusion

Patients who experienced acute LVO moderate and moderate-to-severe ischemic stroke and received loading and maintenance doses of cilostazol within the first 24 h after stroke onset had better clinical outcomes based on recurrent stroke rates and better safety outcomes regarding hemorrhagic transformation of brain infarction and drug-induced peripheral hemorrhagic side effects compared to those who received loading and maintenance doses of clopidogrel.

There were no significant differences between the two groups regarding death due to vascular events and unfavorable mRS after three months of stroke onset.

## Supplementary Information

Below is the link to the electronic supplementary material.

ESM 1 (DOC 219 KB) 

ESM 2 (PDF 82.2 KB)

## Data Availability

The datasets generated during and/or analysed during the current study are available from the corresponding author on reasonable request.

## Declarations

### Guarantor

Mohamed G. Zeinhom is the guarantor of the study.
