# Supplementary material for: Cilostazole versus clopidogrel in acute large-vessel moderate and moderate-to-severe ischemic stroke: a randomized controlled trial
Source: Neurol Sci. 2025 Apr 15;46(8):3973–86. doi: 10.1007/s10072-025-08107-9 (PMC12267345; doi:10.1007/s10072-025-08107-9)
Supplement: Supplementary file 2 — (PDF 82.2 KB) [file 10072_2025_8107_MOESM2_ESM.pdf]

ClinicalTrials.gov PRS
Protocol Registration and Results System

ClinicalTrials.gov Protocol Registration and Results System (PRS) Receipt
Release Date: August 16, 2024

ClinicalTrials.gov ID: NCT06242145

Study Identification

Unique Protocol ID: 00023988

Brief Title: Clopidogrel Versus Cilostazol in Large-vessel Ischemic Stroke

Official Title: Clopidogrel Versus Cilostazol in Large-vessel Ischemic Stroke, a Randomized Controlled Trial

Secondary IDs:

Study Status

Record Verification: August 2024

Overall Status: Completed

Study Start: January 1, 2023 [Actual]

Primary Completion: July 1, 2024 [Actual]

Study Completion: August 1, 2024 [Actual]

Sponsor/Collaborators

Sponsor: Kafrelsheikh University

Responsible Party: Principal Investigator
Investigator: Mohamed G. zeinhom, MD [mgomaa]
Official Title: principal investigator
Affiliation: Kafrelsheikh University

Collaborators:

Oversight

U.S. FDA-regulated Drug: Yes

U.S. FDA-regulated Device: No

Product Exported from U.S.: Yes

U.S. FDA IND/IDE: No

Human Subjects Review: Board Status: Approved
Approval Number: MKSU 50-05-11
Board Name: kafr elsheikh scientific research ethical committee
Board Affiliation: kafr elsheikh university
Phone: 01009625852
Email:
Address:

Data Monitoring: No

FDA Regulated Intervention: Yes

Section 801 Clinical Trial: No

Study Description

Brief Summary: Along with the current clinical trial, the efficacy and safety of a 300 mg loading dose of ticagrelor administered within 24 hours of the first-ever large-vessel ischemic stroke compared to 200 mg cilostazol were assessed through NIHSS, mRS, and possible adverse effects.

Detailed Description: The investigators conducted a single-blinded randomized controlled trial after the ethics committee of the faculty of medicine at Kafr el-Sheik University approved it.

The investigators got written informed consent from all eligible patients or their first order of kin before randomization.

The study will be composed of 2 arms clopidogrel arm, which consisted of 290 patients who received a 300 mg loading dose followed by 75 mg once daily from the 2nd to the 90th day), and the cilostazol arm, consisting of 290 patients who received (a 200 mg loading dose during the first 24 hours of stroke onset followed by 100 mg twice daily from the 2nd day to the 90th day),

Study Procedures:

Every patient in our study will undergo:

clinical workup: History, clinical assessment & NIHSS were recorded on admission, day 7, and the Modified Rankin Scale as a follow-up after one week and 3 months.

Detection of Risk Factors & Profiles:

Echocardiography TTE: in indicated patients ECG Monitoring: daily ECG monitoring will be performed in indicated patients. 3- Carotid Duplex: carotid duplex in indicated patients.

4- ESR & Lipid Profile& liver functions: All will be tested routinely for all patients.

Imaging Follow-UP Non-contrast CT brain on admission Day 2 MRI: after 2 days of admission, all the patients in this study will have a brain MRI (stroke protocol; T1W, T2W, FLAIR, DWI, T2 Echo Gradient, MRA of all intra-cerebral vessels).

CT brain: Any patient with unexplained clinical deterioration at any time throughout his/her hospital stay will be urgently imaged by CT.

Primary End Point:

The primary efficacy outcome was the rate of new stroke at 90 days, and the primary safety outcome was the rate of drug hemorrhagic complications using the PLATO bleeding definition.

• Secondary End Point: The secondary efficacy outcomes were to evaluate the rates of patients who achieved a significant reduction in NIHSS (decrease of four points or more) at the seventh day or discharge compared to baseline, the rates of a favorable outcome with (mRS = 0-2) after one week and after 90 days in a face-to-face interview in the outpatient clinic, rates of a composite of recurrent stroke, myocardial infarction and death due to vascular events

after 90 days of follow-up, while the secondary safety outcome was the rate of treatment-related adverse effects assessed by a follow-up questionnaire

Conditions

Conditions: Ischemic Stroke

Keywords: cilostazol
clopidogrel
large-vessel ischemic stroke

Study Design

Study Type: Interventional

Primary Purpose: Treatment

Study Phase: Phase 3

Interventional Study Model: Parallel Assignment
The investigators will conduct our single-blinded randomized controlled trial, which will contain 2 arms; the clopidogrel arm will receive (a 300 mg loading dose during the first 24 hours of stroke onset, followed by 75 mg daily from the 2nd to the 90th day). The cilostazol arm will receive (a 200mg loading dose during the first 24 hours of stroke onset, followed by 100 mg twice daily from the 2nd to the 90th day).

Number of Arms: 2

Masking: Double (Investigator, Outcomes Assessor)
An independent statistician generated a blocked randomization sequence using computer-generated random numbers; in a one-to-one ratio, participants were randomly assigned to receive either loading doses of clopidogrel or cilostazol by a specially trained and qualified nurse. We prepared Sequentially numbered opaque sealed envelopes and 580 labels for each drug labeled Drug A or B. According to the randomization chart, put them into envelopes numbered 1 to 580. Envelopes were attached to the patient's files. Patients were recruited sequentially and were given enrolment numbers starting from 1, which were mentioned on their files. Files with the same number as the patient enrolment number were opened and the patients were assigned to receive drugs A or B. Drug A included clopidogrel, and Drug B included cilostazol. The statistical analysis was performed by an independent statistician who did not know the treatment protocol of groups A or B.

Allocation: Randomized

Enrollment: 580 [Actual]

Arms and Interventions

Arms	Assigned Interventions
Active Comparator: clopidogrel arm<br>The clopidogrel arm will receive (300 mg loading dose during the first 24 hours of stroke onset, followed by 75 mg daily from the 2nd to the 90th day)	Drug: Clopidogrel tablet<br>Efficacy and safety of a 300mg loading dose of ticagrelor administered within 24 hours of first-ever ischemic stroke followed by 75mg daily for 3 months will be assessed through NIHSS, mRS, duration of hospital stay, new ischemic stroke, and possible adverse effects.<br>Other Names:

Arms	Assigned Interventions
	• group A
Active Comparator: cilostazol arm<br>The cilostazol arm will receive (a 200 mg loading dose during the first 24 hours of stroke onset, followed by 100 mg twice daily from the 2nd day to the 90th day)	Drug: Cilostazol 100 MG<br>Efficacy and safety of 200 mg clopidogrel followed by 100 mg twice daily for 3 months will be assessed through NIHSS, mRS, duration of hospital stay, new ischemic stroke, and possible adverse effects.<br>Other Names:<br>• group B

Outcome Measures

Primary Outcome Measure:

1. rate of new stroke
   Rates of new stroke occur within three months of treatment. The investigators will perform follow-ups of the patient during visits to the outpatient clinic, and brain CT and/ or MRI will be done if there is suspicion of new stroke.

   [Time Frame: 90 days]
2. Rate of drug-related hemorrhagic complications
   the rate of drug hemorrhagic complications which was evaluated using the PLATO bleeding definition which classified hemorrhagic complications into three types as follows: Major bleeding which had one or more of the following criteria: fatal bleeding, intracranial, intrapericardial, bleeding associated with reduction of hemoglobin > 3-5 g/dl, bleeding required transfusion of two to four units whole blood or PRBCs, bleeding produced hypovolemic shock or severe hypotension that required pressor or surgery; Minor bleeding that required medical intervention to stop or treat bleeding: Minimal bleeding: any bleeding that did not require intervention or treatment such as bruising, bleeding gums, oozing from injection sites.

   [Time Frame: 90 days]

Secondary Outcome Measure:

3. Value of National Institute of Health Stroke Scale (NIHSS) after one week
   NIHSS is a tool used by healthcare providers to objectively quantify the impairment caused by a stroke and aid in planning post-acute care disposition.

   It ranges from 0 to 42; the lower the score, the better the stroke condition. The improvement will be counted only if there is a decrease in NIHSS score by four points or more within one week of stroke onset.

   [Time Frame: 7 days]
4. Value of Modified Rankin Scale (mRS) at one week
   mRS Measures the degree of disability or dependence in the daily activities of people who have suffered a stroke or other causes of neurological disability; its value ranges from 0 to 6; the lower the score, the better the stroke outcome. A favorable stroke outcome is considered with mRS value equals two or less

   [Time Frame: 7 days]
5. value of Modified Rankin Scale(mRS) at three months
   mRS Measures the degree of disability or dependence in the daily activities of people who have suffered a stroke or other causes of neurological disability; its value ranges from 0 to 6; the lower the score, the better the stroke outcome. A favorable stroke outcome is considered with mRS value equals two or less.

   [Time Frame: 3 months]
6. rate of composite recurrent stroke, myocardial infarction, and death due to vascular events
   rates of new stroke, TIA, myocardial infarction, or death from vascular events within three months of treatment the investigators will perform follow-ups of the patient during visits to the outpatient clinic and perform needed investigations such as brain imaging, Electrocardiography, arterial and venous duplex ultrasound imaging

   [Time Frame: 3 months]
7. rate of drug adverse effects

Drug adverse effects: all side effects related to the drugs of our study will be reported

[Time Frame: 90 days]

Eligibility

Minimum Age: 18 Years

Maximum Age: 75 Years

Sex: All

Gender Based: No

Accepts Healthy Volunteers: No

Criteria: Inclusion Criteria:

- the investigators included both genders with eligible ages ranging between 18-75 years, with the first-ever presentation with acute large-vessel ischemic stroke who received antiplatelet treatment within the first 24 hours of the onset of ischemic stroke. Patients with previous transient ischemic attacks (TIA) were not excluded from the study. Patients are not eligible for rt-PA treatment

Exclusion Criteria:

- The investigators excluded patients who had not been followed up on for 90 days after enrollment, those with NIHSS ≤ 3 or ≥ 25 or who had rapidly resolving symptoms before imaging results, and patients with a known history of persistent or recurrent CNS pathology (e.g., epilepsy, meningioma, multiple sclerosis, history of head trauma with a residual neurological deficit).

The investigators excluded patients who had clinical seizures at the onset of their stroke, as well as those who had symptoms of any major organ failure, active malignancies, or an acute myocardial infarction within the previous six weeks, and those who were on warfarin, regular ticagrelor during the week before admission, or chemotherapy within the previous year.

The investigators excluded patients with active peptic ulcers, GIT surgery, bleeding history within the last year, and those with a history of major surgery within the last three months.

The investigators ruled out of our trial patients who had a known allergy to the study drugs and those with INR > 1.4 or P.T. >18 or blood glucose level < 50 or > 400 mg/DL or blood pressure < 90/60 or > 185/110 mmHg on admission or Platelets < 100,000.

The investigators excluded pregnant and lactating patients and those with stroke due to venous thrombosis and stroke following cardiac arrest or profuse hypotension ineligible for our trial.

Patients with contraindications to the study drugs were excluded.

Contacts/Locations

Central Contact Person: mohamed G Zeinhom, MD
Telephone: 2001009606828
Email: mohamed_gomaa@med.kfs.edu.eg

Central Contact Backup: sherihan R. ahmed, MD
Telephone: 2001113432342
Email: sherihanrezk2016@gmail.com

Study Officials: mohamed G. Zeinhom, MD
Study Director
neurology department kafr el-sheikh university

Locations: **Egypt**
kafr elsheikh university hospital
Kafr Ash Shaykh, Egypt, 33155
Contact: mohamed G. Zeinhom, MD 2001009606828
mohamed_gomaa@med.kfs.edu.eg
Contact: sherihan R ahmed, MD 2001007481842
sherihanrezk2016@gmail.com

IPDSharing

Plan to Share IPD: No
All the data that support the findings of this research will be available from the corresponding author M. Zeinhom upon reasonable request.

References

Citations: **[Study Results]** Paciaroni M, Ince B, Hu B, Jeng JS, Kutluk K, Liu L, Lou M, Parfenov V, Wong KSL, Zamani B, Paek D, Min Han J, Del Aguila M, Girotra S. Benefits and Risks of Clopidogrel vs. Aspirin Monotherapy after Recent Ischemic Stroke: A Systematic Review and Meta-Analysis. Cardiovasc Ther. 2019 Dec 1;2019:1607181. doi: 10.1155/2019/1607181. eCollection 2019. PubMed 31867054

[Study Results] Meyer DM, Albright KC, Allison TA, Grotta JC. LOAD: a pilot study of the safety of loading of aspirin and clopidogrel in acute ischemic stroke and transient ischemic attack. J Stroke Cerebrovasc Dis. 2008 Jan-Feb;17(1):26-9. doi: 10.1016/j.jstrokecerebrovasdis.2007.09.006. PubMed 18190818

[Study Results] Gachet C, Stierle A, Cazenave JP, Ohlmann P, Lanza F, Bouloux C, Maffrand JP. The thienopyridine PCR 4099 selectively inhibits ADP-induced platelet aggregation and fibrinogen binding without modifying the membrane glycoprotein IIb-IIIa complex in rat and in man. Biochem Pharmacol. 1990 Jul 15;40(2):229-38. doi: 10.1016/0006-2952(90)90683-c. PubMed 2375765

Links:

Available IPD/Information:
